# Evaluation of the heterogeneous tissue distribution of erlotinib in lung cancer using matrix-assisted laser desorption ionization mass spectrometry imaging

**DOI:** 10.1038/s41598-017-13025-8

**Published:** 2017-10-03

**Authors:** Yukari Tsubata, Mitsuhiro Hayashi, Ryosuke Tanino, Hiroaki Aikawa, Mayu Ohuchi, Kenji Tamura, Yasuhiro Fujiwara, Takeshi Isobe, Akinobu Hamada

**Affiliations:** 1 Division of Medical Oncology and Respiratory Medicine, Department of Internal Medicine, Shimane University, School of Medicine, 89-1 Enya-cho, Izumo, Shimane 693-8501 Japan; 2 Division of Molecular Pharmacology, National Cancer Center Research Institute, National Cancer Center, 5-1-1 Tsukiji, Chuo-ku Tokyo, 104-0045 Japan; 3 Division of Clinical Pharmacology and Translational Research, Exploratory Oncology Research and Clinical Trial Center, National Cancer Center, 5-1-1 Tsukiji, Chuo-ku Tokyo, 104-0045 Japan; 4 Department of Breast and Medical Oncology, National Cancer Center Hospital, National Cancer Center, 5-1-1 Tsukiji, Chuo-ku Tokyo, 104-0045 Japan

## Abstract

Although drug distribution in tumor tissues has a significant impact on efficacy, conventional pharmacokinetic analysis has some limitations with regard to its ability to provide a comprehensive assessment of drug tissue distribution. Erlotinib is a tyrosine kinase inhibitor that acts on the epidermal growth factor receptor; however, it is unclear how this drug is histologically distributed in lung cancer. We used matrix-assisted laser desorption/ionization mass spectrometry imaging (MALDI-MSI) and liquid chromatography-tandem mass spectrometry (LC-MS/MS) to analyze erlotinib distribution in the tumor and normal lung tissues of a mouse xenograft model and patient with non-small cell lung cancer. LC-MS/MS showed that the erlotinib tissue concentration in the xenograft tumor tissue was clearly lower than that in the normal tissue at the time of maximum blood concentration. MALDI-MSI showed the heterogeneous distribution of erlotinib at various levels in the murine tissues; interestingly, erlotinib was predominantly localized in the area of viable tumor compared to the necrotic area. In the patient-derived tissue, MALDI-MSI showed that there were different concentrations of erlotinib distributed within the same tissue. For drug development and translational research, the imaging pharmacokinetic study used the combination of MALDI-MSI and LC-MS/MS analyses may be useful in tissues with heterogeneous drug distribution.

## Introduction

The measurement of drug concentrations in target tissues plays a critical role in determining the appropriate drug dosage and evaluating the therapeutic window in the development of anticancer drugs^1^. Improvements are still needed in preclinical evaluation assays, because treatment failure is often caused by lack of efficacy and unacceptable clinical safety profiles of investigational compounds^2–4^. Liquid chromatography-tandem mass spectrometry (LC-MS/MS), which is generally used to measure drug concentrations in blood or tissues, provides accurate quantitative information; however, it has not been of substantial benefit in evaluating drug distribution in tissues. LC-MS/MS requires liquid samples, so the homogenization of tissue samples is needed to measure drug concentrations in tissues^5^.

It was recently suggested that mass spectrometry imaging (MSI) may be used to study tissues with heterogeneous morphology^6–8^. Erlotinib is a tyrosine kinase inhibitor that works by inhibiting epidermal growth factor receptor (EGFR); a mutation in *EGFR* confers an increased response to this drug^9,10^. However, erlotinib distribution in patient tumor tissue has never been clearly shown by histology. It seems to be useful that the innovative MSI technique translate into the visualization of erlotinib distribution in microscopic level as the no-labeling technique.

The aim of this study was to use a combination of matrix-assisted laser desorption ionization (MALDI)-MSI and liquid chromatography-tandem mass spectrometry (LC-MS/MS) to evaluate the distribution of erlotinib in the tumor and normal lung tissues of a mouse xenograft model and patient with non-small cell lung cancer.

## Results

**Figure 1:**
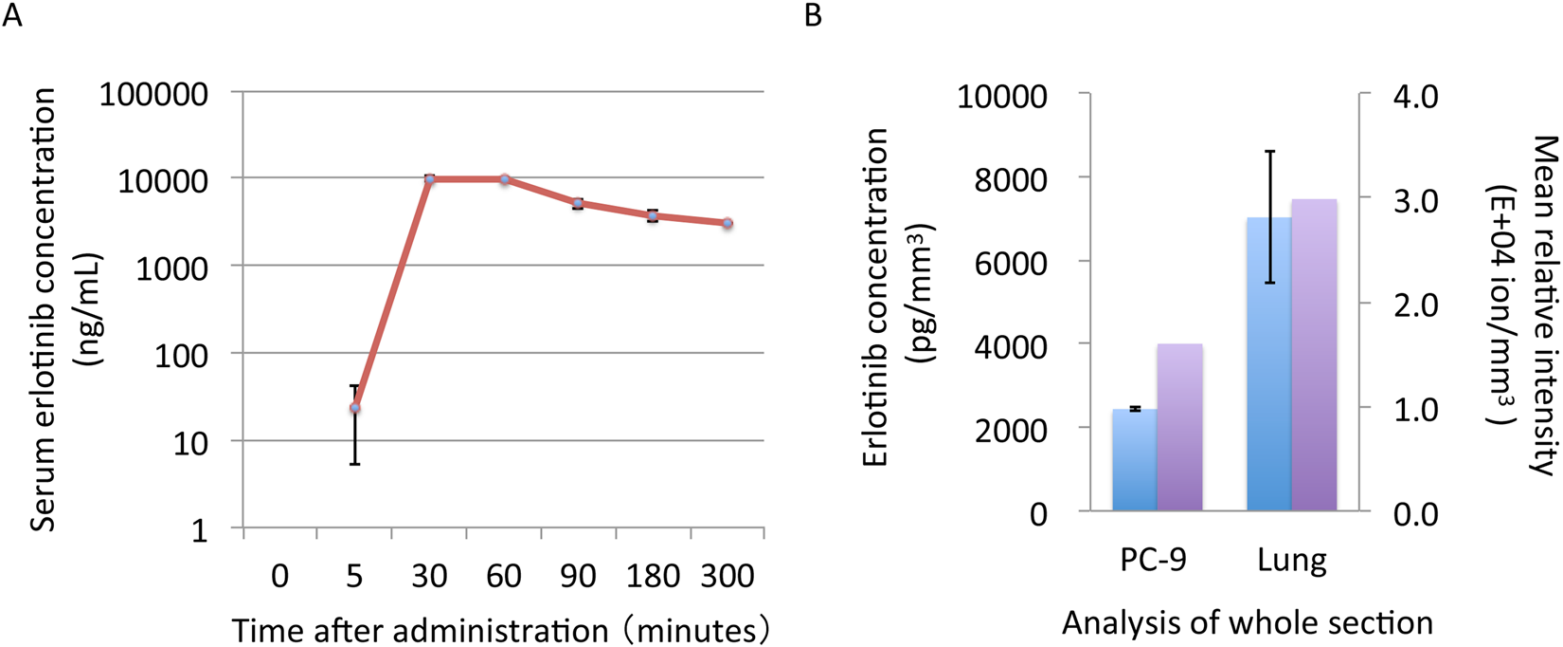
Serum and tissue erlotinib concentrations in the mouse xenograft model (**A**) Serum erlotinib concentration by liquid chromatography-tandem mass spectrometry (**B**) Erlotinib concentrations in the tissue sections was compared between tumor and normal lung tissues. Blue bar indicates erlotinib concentration by liquid chromatography-tandem mass spectrometry. Purple bar indicates erlotinib ion intensity per section by matrix-assisted laser desorption ionization mass spectrometry imaging. Error bar = standard deviation of replicate samples.

In the pharmacokinetics analysis of the PC-9 xenograft model, the time of maximum serum concentration of erlotinib (T_max_) was 60 min after oral administration of 25 mg/kg doses (Fig. 1A); thus, we decided to use this time point for subsequent erlotinib analyses. Unexpectedly, the erlotinib concentration in the tumor section was approximately one-third that of the normal lung section, as determined by LC-MS/MS analysis (mean ± standard deviation: tumor section, 2438 ± 54 pg/mm^3^ vs. normal lung section, 7026 ± 1567 pg/mm^3^) (Fig. 1B).

**Figure 2:**
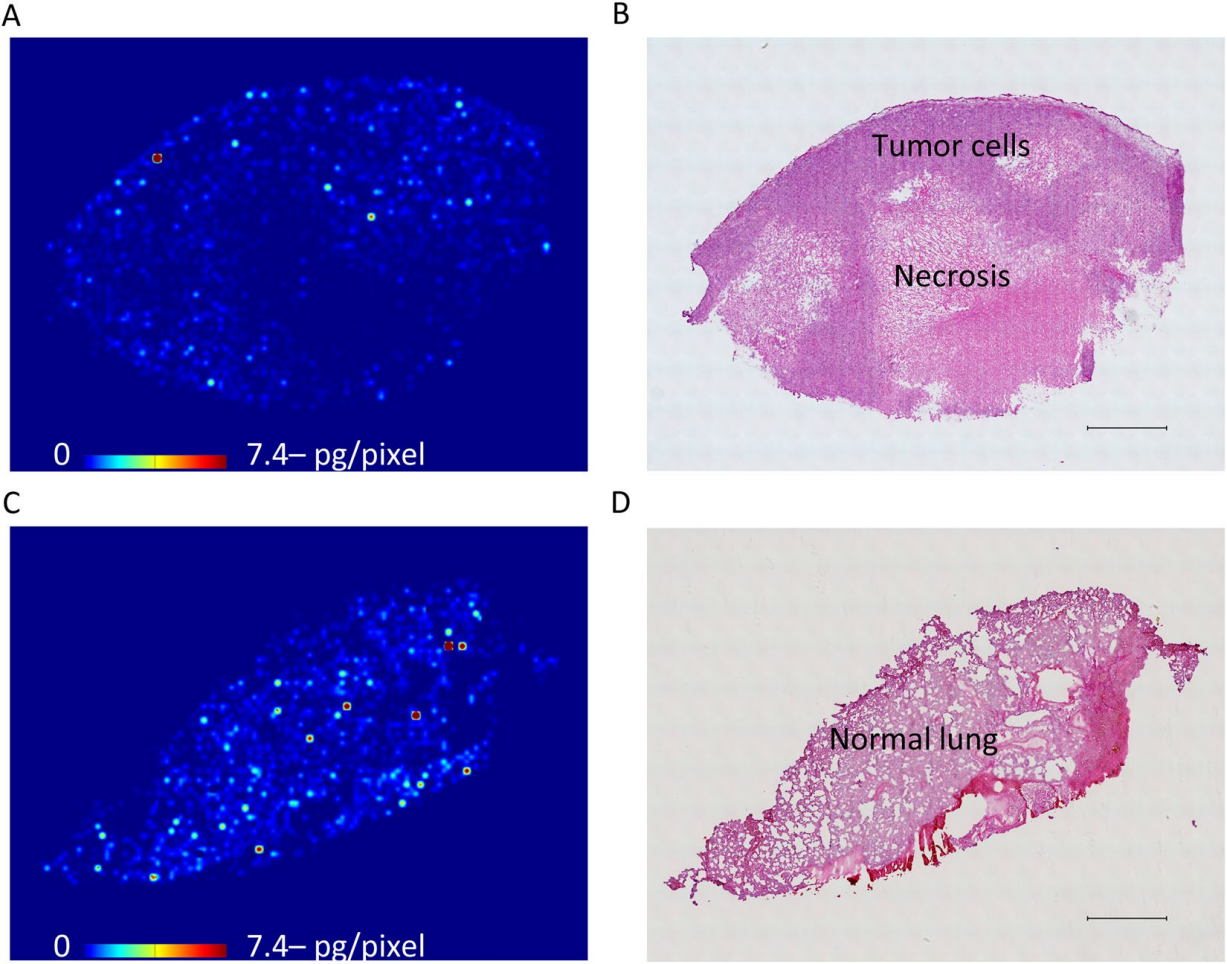
Heterogeneous erlotinib distribution in mouse tissue sections at T_max_ using matrix-assisted laser desorption/ionization mass spectrometry imaging (**A**) Molecular image of erlotinib in the tumor sections by using matrix-assisted laser desorption/ionization mass spectrometry imaging. Scale bar indicates erlotinib quantity per pixel, and upper limit of the scale bar indicates more than 7.4 pg/pixel erlotinib. Molecular images were acquired at a step size of 60 μm. (**B**) Hematoxylin and eosin staining of the tumor section was performed after acquiring the mass spectrometry image. Scale bar = 1 mm. (**C**) Molecular image of erlotinib in the normal mouse lung tissue section (**D**) Hematoxylin and eosin staining of the normal lung tissue section after acquiring the mass spectrometry image. Scale bar = 1 mm.

**Figure 3:**
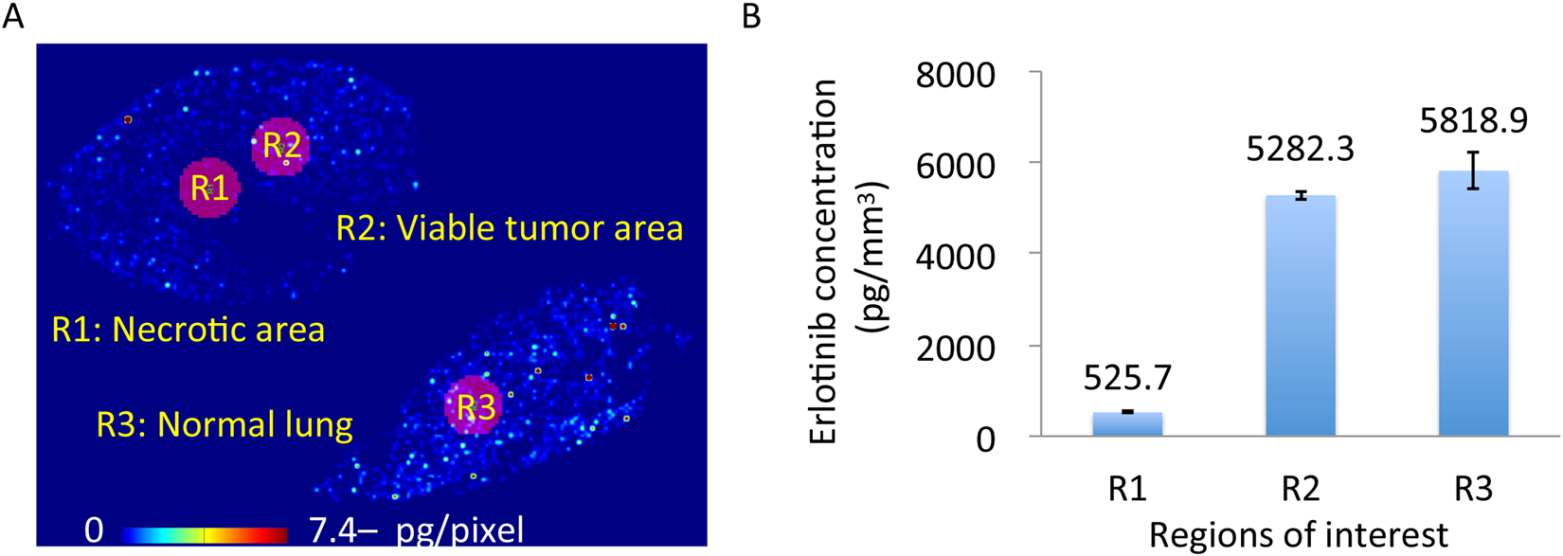
Comparison of relative erlotinib concentrations among necrotic, viable tumor, and normal lung regions in the mouse xenograft model (**A**) Erlotinib tissue distribution by matrix-assisted laser desorption/ionization mass spectrometry imaging and regions of interest are shown. (**B**) Relative erlotinib concentrations were estimated from the mass spectrometry images and liquid chromatography-tandem mass spectrometry using serial sections, and were compared among necrotic, viable tumor, and normal mouse lung regions in the mouse xenograft model. Error bar = standard deviation of replicate analysis for each region of interest.

Next, we evaluated the tissue distribution of erlotinib by MALDI-MSI, which showed the more heterogeneous distribution of erlotinib in the tumor section compared to the normal lung section (Fig. 2, Supplementary Fig. 1). Erlotinib was predominantly localized in the area of viable tumor compared to the necrotic area (Fig. 2A,B). When we compared the relative erlotinib concentration, quantitated using both MALDI-MSI and LC-MS/MS, we found concentrations of 526 ± 12 pg/mm^3^ in the necrotic area, 5282 ± 91 pg/mm^3^ in the viable tumor area, and 5819 ± 394 pg/mm^3^ in the normal lung area (Fig. 3). No notable difference in erlotinib concentration was observed between the viable tumor and normal lung areas in the xenograft model.

**Figure 4:**
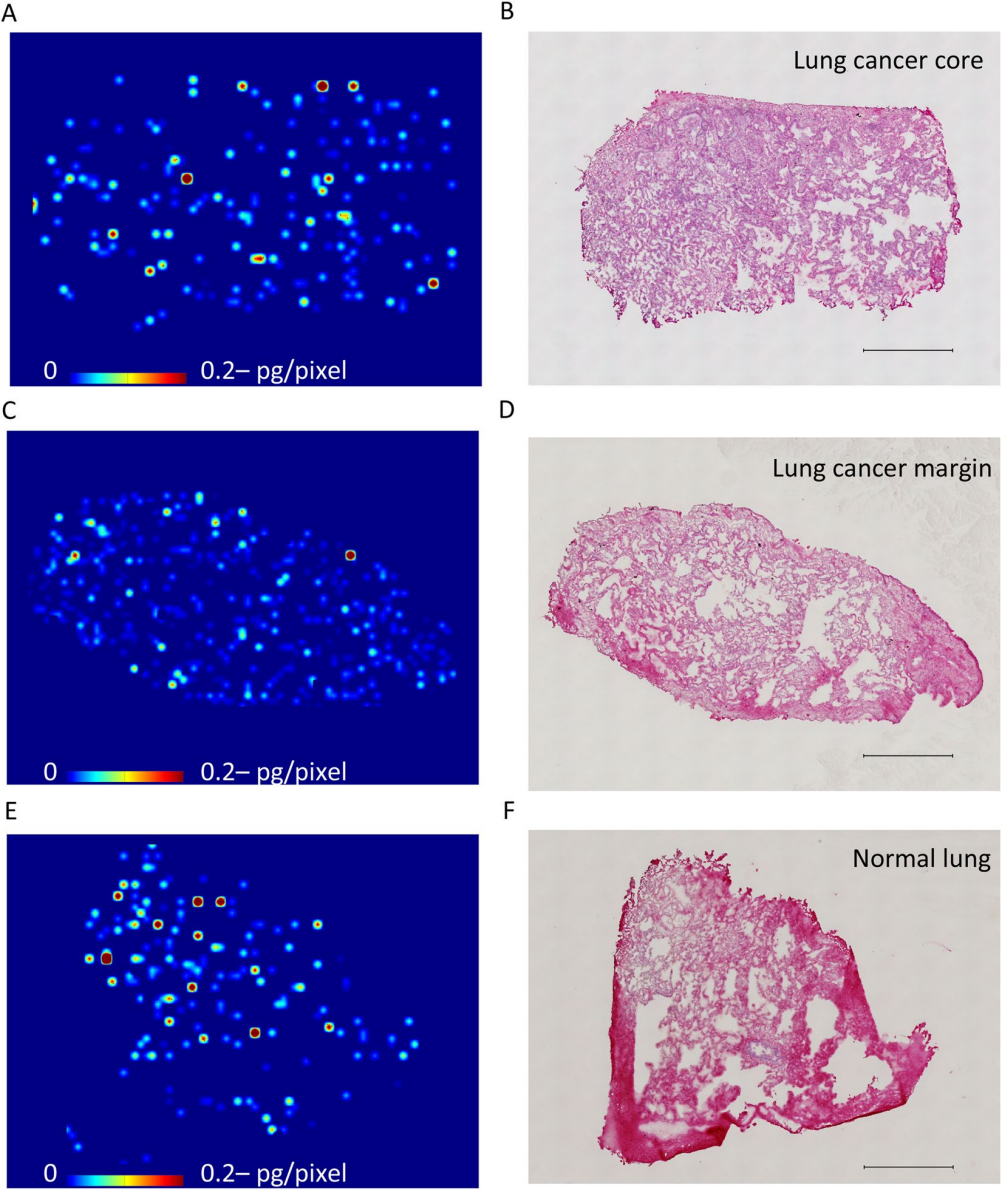
Erlotinib tissue distribution in lung cancer with the epidermal growth factor receptor L858R mutation (**A**,**C**,**E**) Molecular images of heterogeneous erlotinib distribution at 6 h after single administration of 150 mg in the lung cancer core, marginal region, and normal lung tissues, respectively. Scale bar indicates erlotinib quantity per pixel, and upper limit of the scale bar indicates more than 0.2 pg/pixel erlotinib. Molecular images were acquired at a step size of 60 μm. (**B**,**D**,**F**). Hematoxylin and eosin staining of the sections after acquiring the mass spectrometry image. Scale bar = 1 mm.

Finally, we analyzed human lung cancer samples 6 h after the single oral administration of 150 mg/body, which is the T_max_ in humans^11^. LC-MS/MS analysis showed that there were no apparent differences in erlotinib tissue concentrations among the lung cancer core, marginal region, and normal lung regions (140 ± 25 pg/mm^3^, 125 ± 2 pg/mm^3^, and 159 ± 37 pg/mm^3^, respectively). MALDI-MSI revealed the same degree of heterogeneous erlotinib distribution in these tissue sections (Fig. 4) (Supplementary Fig. 3). We microdissected representative pieces from each section and evaluated erlotinib concentrations by LC-MS/MS. The analysis showed no remarkable difference among pieces from the human lung cancer core, marginal region, and normal lung region (102 pg/mm^3^, 124 pg/mm^3^, and 122 pg/mm^3^, respectively) (Supplementary Fig. 4).

## Discussions

The most important goal of this study was to visualize the intratumoral distribution of erlotinib in both a mouse xenograft model and human lung cancer tissues using MALDI-MSI. Although we have not been able to determine if the drug actually reached the tumor cells by the conventional pharmacokinetics study, it may be possible to track this occurrence in tissue sections in the future. Radioisotope and fluorescent labeling techniques are often used in drug tissue distribution studies; however, they have significant drawbacks including the high label densities required, the introduction of possible artifacts due to the labels themselves, and the fact that some macromolecular structures are difficult to label.

In this study, conventional LC-MS/MS showed that in the xenograft model, the tissue concentration of erlotinib was higher in the normal lung than in the tumor, but was similar in patient-derived normal and tumor tissues, indicating that there was a discrepancy in the results obtained in preclinical and clinical settings. On the other hand, MALDI-MSI showed that erlotinib was less localized in the tumor necrosis region and was at present at similar levels in the viable xenograft tumor and normal lung tissue. Thus, conventional LC-MS/MS was unable to show the heterogeneous distribution of erlotinib^5^. However, MALDI-MSI technique resolved the inconsistency between the preclinical and clinical results. Additional studies with a cohort of patients and range of time points are needed to confirm the usefulness of MALDI-MSI in evaluating drug distribution. In addition, improving spatial resolution will lead to high-precision results^12^. Finally, the precise observation of intratumoral drug distribution will be an important feature of innovative drug development, as well as for predicting drug efficacy and safety^13^.

In conclusion, this pharmacokinetic study using MALDI-MSI showed the heterogeneous tissue distribution of erlotinib in mouse and patient tumors. No uniform concentration of erlotinib was observed even in the same tissue, demonstrating the usefulness of this technique for evaluating drug tissue distribution. Such drug tissue distribution studies by MSI may contribute to the reuse of drugs that were previously underestimated by LC-MS/MS analysis, and may also reveal the usefulness of tissue therapeutic drug monitoring in translation cancer research.

## Methods

### Animal models

Animal studies were carried out according to the Guideline for Animal Experiments, drawn up by the Committee for Animal Experimentation of Shimane University, which meet the ethical standards required by the law and the guidelines about experimental animals in Japan. All animal experimental protocols were approved by the Animal Experiment Committee of the Shimane University (Permit Number: IZ27-76). PC-9 human lung cancer cells were subcutaneously implanted into BALB/c *nu/nu* mice. After the tumors had grown (day 28), erlotinib was orally administered at a dose of 25 mg/kg, and tissue and serum samples were collected at 5 to 300 min after the administration of erlotinib. Tissue samples of the dissected tumor and lung were flash-frozen in liquid nitrogen. All of the tissues and sera were stored at −80 °C until subsequent use.

### Clinical samples

An 80-year-old nonsmoking woman exhibited a 35 × 25 mm ground-glass nodule in the left upper lobe (S1 + 2) of the lung on computed tomography during a medical check-up (Supplementary Fig. 2). She was diagnosed with non-small cell lung cancer (cT2aN0M0, stage IB) harboring an activating EGFT mutation (exon21 L858R). This trial was registered with UMIN 000009745 (Jan 10, 2013). The ethics committee of Shimane University approved the study protocol, and the study was conducted in accordance with the principles of the Declaration of Helsinki. We received written informed consent for the oral administration of erlotinib prior to surgery. The patient received erlotinib at a dose of 150 mg. After 6 h, tumor and normal tissues were surgically resected. Then we sampled the flash-frozen tissues of lung cancer (tumor core and margin) and normal lung to analyze erlotinib distribution. The patient did not experience any side effects, and was released from the hospital 12 days after surgery.

### Tissue slice preparation

The tissue was sliced into 8 μm thick slices with a Cryomicrotome (Leica CM 1950, Tokyo, Japan) into at least three serial sections. The first and third sections were used for LC-MS/MS measurement of erlotinib concentration in the tissue, whereas the second section was placed on indium tin oxide-coated (ITO) glass slides (Matsunami Glass Ind., Ltd., Tokyo, Japan) and used for MALDI-MSI analysis of intra-tissue erlotinib distribution and for calculating the area of the tissue sections using the BZ-X710 microscope (Keyence, Itasca, IL, USA). The additional 16 serial sections of the tissue were used for LC-MS/MS measurement of erlotinib concentration in the small pieces that were cut by laser microdissection (Leica LMD 6500). Due to the limited clinical specimens, multiple analysis of laser microdissection was difficult in this study.

### Mass spectrometry analysis

Details of the MS methods are provided in the Supplementary Information section. Briefly, erlotinib concentrations in the plasma and tissue homogenates were measured using a QTRAP 4500 mass spectrometer (AB SCIEX, Framingham, MA, USA) coupled to a Nexera ×2 HPLC system (Shimadzu, Kyoto, Japan). The detection of erlotinib distribution in the tissues was performed using an iMScope (Shimadzu)^14^.

## Electronic supplementary material

Supplementary information

Clinical trial protocol
