# Supplementary material for: Evaluation of the heterogeneous tissue distribution of erlotinib in lung cancer using matrix-assisted laser desorption ionization mass spectrometry imaging
Source: Sci Rep. 2017 Oct 3;7:12622. doi: 10.1038/s41598-017-13025-8 (PMC5626687; doi:10.1038/s41598-017-13025-8)
Supplement: Supplementary file 1 — Supplementary information [file 41598_2017_13025_MOESM1_ESM.pdf]

Evaluation of the heterogeneous tissue distribution of erlotinib in lung cancer using matrix-assisted laser desorption ionization mass spectrometry imaging

Yukari Tsubata[1)], Mitsuhiro Hayashi[2,3)], Ryosuke Tanino[1)], Hiroaki Aikawa[3)], Mayu Ohuchi[2)], Kenji Tamura[4)], Yasuhiro Fujiwara[4)], Takeshi Isobe[1)], Akinobu Hamada[2,3) *]

1) Division of Medical Oncology and Respiratory Medicine, Department of internal medicine, Shimane University, School of Medicine, 89-1 Enya-cho, Izumo, Shimane, Japan 693-8501

2) Division of Molecular Pharmacology, National Cancer Center Research Institute, National Cancer Center, 5-1-1 Tsukiji, Chuo-ku, Tokyo 104-0045, Japan.

3) Division of Clinical Pharmacology and Translational Research, Exploratory Oncology Research and Clinical Trial Center, National Cancer Center, 5-1-1 Tsukiji, Chuo-ku, Tokyo 104-0045, Japan.

4) Department of Breast and Medical Oncology, National Cancer Center Hospital, National Cancer Center, 5-1-1 Tsukiji, Chuo-ku, Tokyo 104-0045, Japan.

Corresponding author:

Akinobu Hamada, Ph.D.

Division of Molecular Pharmacology, National Cancer Center Research Institute, National Cancer Center, 5-1-1 Tsukiji, Chuo-ku, Tokyo 104-0045, Japan.

Email: akhamad@ncc.go.jp

Supplementary Information

LC-MS/MS

Erlotinib was extracted from mouse serum by solid phase extraction using Oasis HLB 96-well plates (10 mg sorbent per well; Waters, Milford, MA, USA), and calibration standards ranging from 5 to 5000 ng/mL were prepared from standard solutions using a mouse serum pool (Sigma) for the measurement of serum erlotinib concentrations. Erlotinib-d6 was used as the internal standard (IS). Mouse tissue sections and patient samples were homogenized with 100 μL 30% acetonitrile, 10% isopropanol, and 0.1% formic acid (FA) for 1 min, followed by centrifugation at 120,000 x g for 10 min at 4°C. A total of 20 μL supernatant and internal standard, 260 μL 50% methanol, and 0.1% FA were used for the quantitation of erlotinib with calibration standards ranging from 0.1 to 100 ng/mL. Chromatographic separation was performed using an XBridge C18 HPLC column (2.1 x 5.0 mm, 3.5 μm; Waters) maintained at 40°C. Mobile phases A and B consisted of 0.1% FA aqueous solution and acetonitrile containing 0.1% FA, respectively. Separation was performed using a gradient elution at a flow rate of 0.2 mL/min with a Nexera X2 series (Shimadzu). The gradient elution of the two mobile phase systems with 0.1% FA aqueous (A) and acetonitrile containing 0.1% FA (B) was as follows: 20–65% B for 0–4.0 min, 65–90% B for 4.0–4.1 min, 90% B for 4.1–5.1 min, and 90–20% B for 5.1–5.2 min, with a re-equilibrium time of 3 min. The column was equilibrated with the mobile phase for

10 min, with a run time of 6 min for each 20 μL injection volume. Quantitation was conducted by selected reaction monitoring on a QTRAP4500 mass spectrometer (AB SCIEX) with electrospray ionization in the positive mode. The optimized electrospray ionization parameters were as follows: ion source temperature, 600°C; curtain gas, setting 50; nebulizing gas (GS1), setting 70; turbo-ionspray gas (GS2), setting 80; ionspray voltage, 5000 V; declustering potential, 6 V; and collision energy, 43V. The selected reaction monitoring transitions were m/z 394.0 to 336.0 for erlotinib and m/z 400.0 to 338.9 for erlotinib-d6. The dwell time was 500 ms for each transition channel. All data were acquired and analyzed using Analyst 1.6.1 software (AB SCIEX).

MALDI-MSI

Cyano-4-hydroxy-cinnamic acid (CHCA; Sigma) was first coated on an ITO glass slide using a vacuum sublimation apparatus (SVC-700TMSG/7PS80; SANYU Electron, Tokyo, Japan) at 250°C for 8 min, after which 10 mg/mL CHCA solution, containing 30% acetonitrile, 10% isopropanol, and 0.1% FA was sequentially sprayed on the slide using a sprayer (PS270; GSI Creos Corp., Tokyo, Japan) in the following 10 spraying cycle conditions: the first three cycles were for 3 s at 90 s intervals and the next seven cycles were for 1 s at 30 s intervals. To evaluate the ionization efficiency of erlotinib on the tissue sections, erlotinib-d6 was used as the internal standard for the CHCA solution (2 μg/mL). MALDI-MSI of the tissue sections was

performed using an iMScope (Shimadzu) consisting of an optical microscope and a quadrupole ion trap time-of-flight analyzer with an atmospheric pressure MALDI source. The experimental condition was optimized to obtain the best signal-to-noise ratio and reproducibility. MSI data from the tissue sections were analyzed at a spatial resolution of 60 μm in the positive ion mode. Mass spectra were obtained with 150 (50 x 3) laser shots at a mass range of m/z 100.0–396.0 for erlotinib and m/z 100.0–405.0 for erlotinib-d6. The other optimized laser parameters were as follows: strength, setting 50; spot size, setting 2; and frequency, 1,000 Hz. The optimized detector parameters were as follows: sample voltage, 3.5 kV; detector voltage, 2.1 kV; MS/MS energy, 50%; gas volume, 50%; isolation time, 20 ms; and collision-induced dissociation time, 30 m. The optimal transitions of erlotinib and erlotinib-d6 used for MS/MS analysis were m/z 394.10 ± 3.00 to 336.14 ± 0.02 and m/z 400.00 ± 1.5 to 339.16 ± 0.02, respectively. The erlotinib-d6 image was acquired with 30 μm sift from the erlotinib analyzed pixel. Raw molecular images were visualized using MS imaging software (Imaging MS Solution, version 1.20, Shimadzu) for erlotinib and erlotinib-d6 (internal standard) images with an absolute ion intensity scale. Biomap version 3.8.0.4 (Novartis Institutes for BioMedical Research, Basel, Switzerland) was used to display the erlotinib molecular images (divided images of raw erlotinib images by the internal standard). Scale bar of the Biomap images was converted from absolute ion intensity to concentration using the LC-MS/MS results of the serial

sections. MS experiments were performed in part by Shimadzu Techno-Research (Tokyo, Japan).

Supplementary Figures and Figure Legends

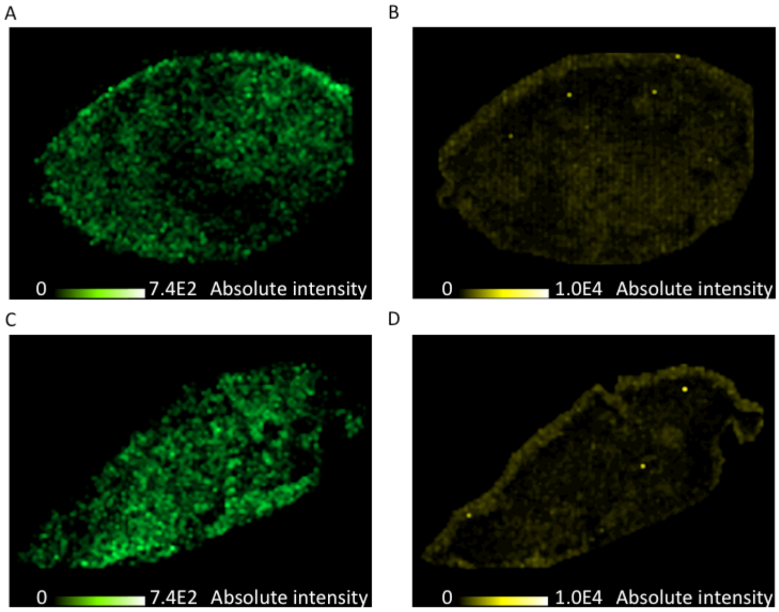

Figure S1. Raw and internal standard images of erlotinib distribution in the mouse tissues by matrix-assisted laser desorption/ionization mass spectrometry imaging

(A) Raw molecular image of erlotinib distribution in the xenograft tumor section using matrix-assisted laser desorption ionization mass spectrometry imaging. Scale bar indicates absolute ion intensities of erlotinib. (B) Molecular image of the exogenous internal standard, erlotinib-d6, in the same tissue section to indicate the basal ionization efficiency of erlotinib in the tissue. (C, D) Raw images of erlotinib and the internal standard in the normal lung section, respectively. Molecular images were acquired at a step size of 60 μm, which generated optimal transitions of erlotinib and erlotinib-d6 at m/z values of 394.10 ($[M+H]^+$) ± 3.00 to 336.15 ± 0.05 and m/z values 400.10 ($[M+H]^+$) ± 1.50 to 339.15 ± 0.05, respectively.

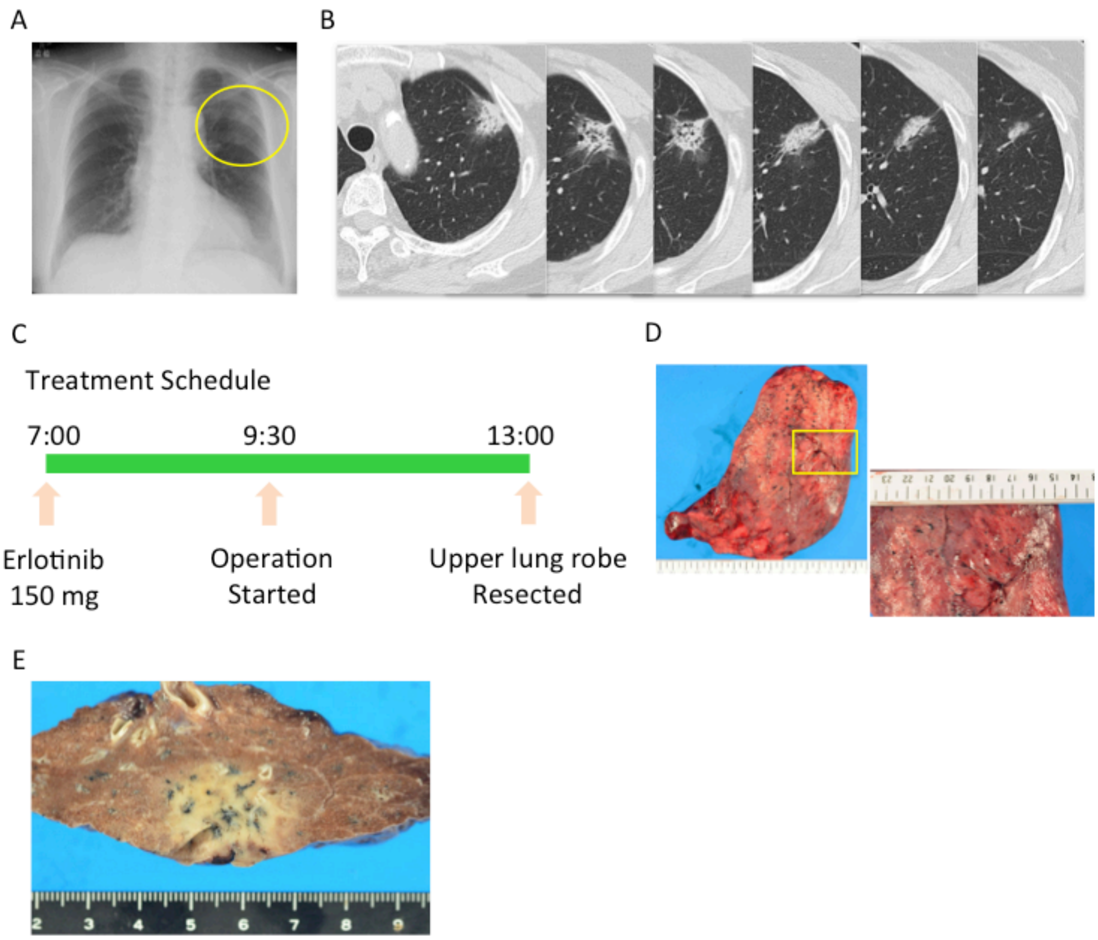

Figure S2. Clinical information on lung cancer tissues

(A) Chest X-ray of the patient (B) Computed tomography showed typical images of non-small cell lung cancer. (C) Schedule of erlotinib oral administration and surgery (D) Flash frozen samples for mass spectrometry analyses were collected from the surgical specimen. (E) The formalin-fixed specimen clearly shows the lung cancer lesion.

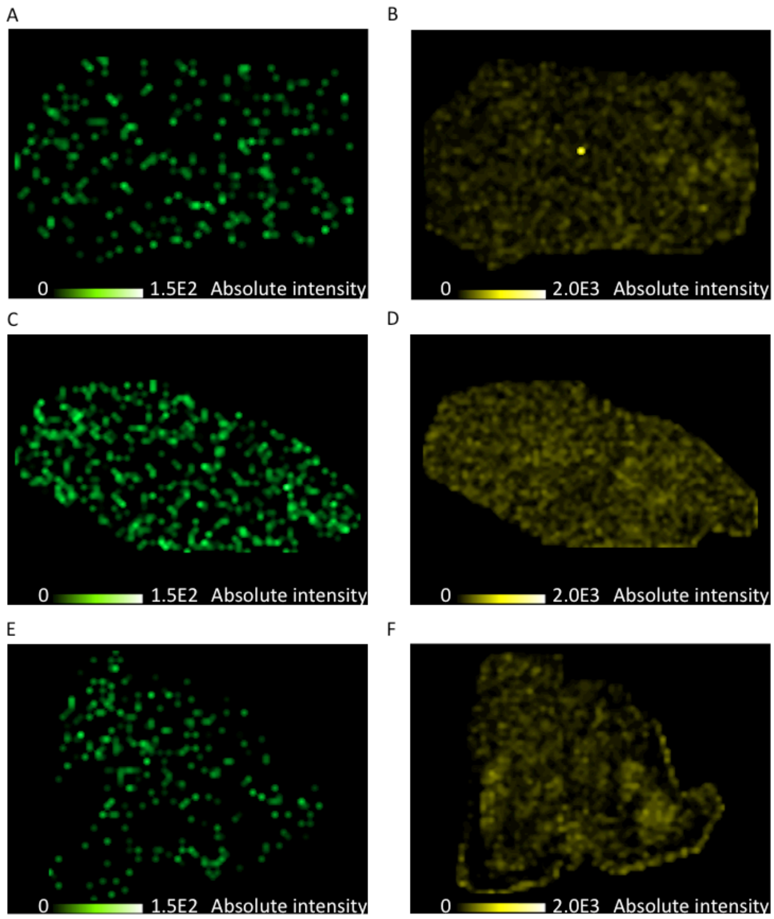

Figure S3. Raw and internal standard images of erlotinib tissue distribution in lung cancer with the epidermal growth factor receptor L858R mutation

(A, C, E) Raw molecular image of erlotinib using matrix-assisted laser desorption ionization mass spectrometry imaging in the lung cancer core, marginal region, and normal lung tissues, respectively. Scale bar indicates absolute ion intensities of erlotinib. (B, D, F) Molecular image of the exogenous internal standard, erlotinib-d6, in the same sections of the raw molecular images. Molecular images were acquired at a step size of 60 μm, which generated optimal transitions of erlotinib and erlotinib-d6 at m/z values of 394.10 ($[M+H]^+$) ± 3.00 to 336.15 ± 0.05 and m/z 400.10 ($[M+H]^+$) ± 1.50 to 339.15 ± 0.05, respectively.

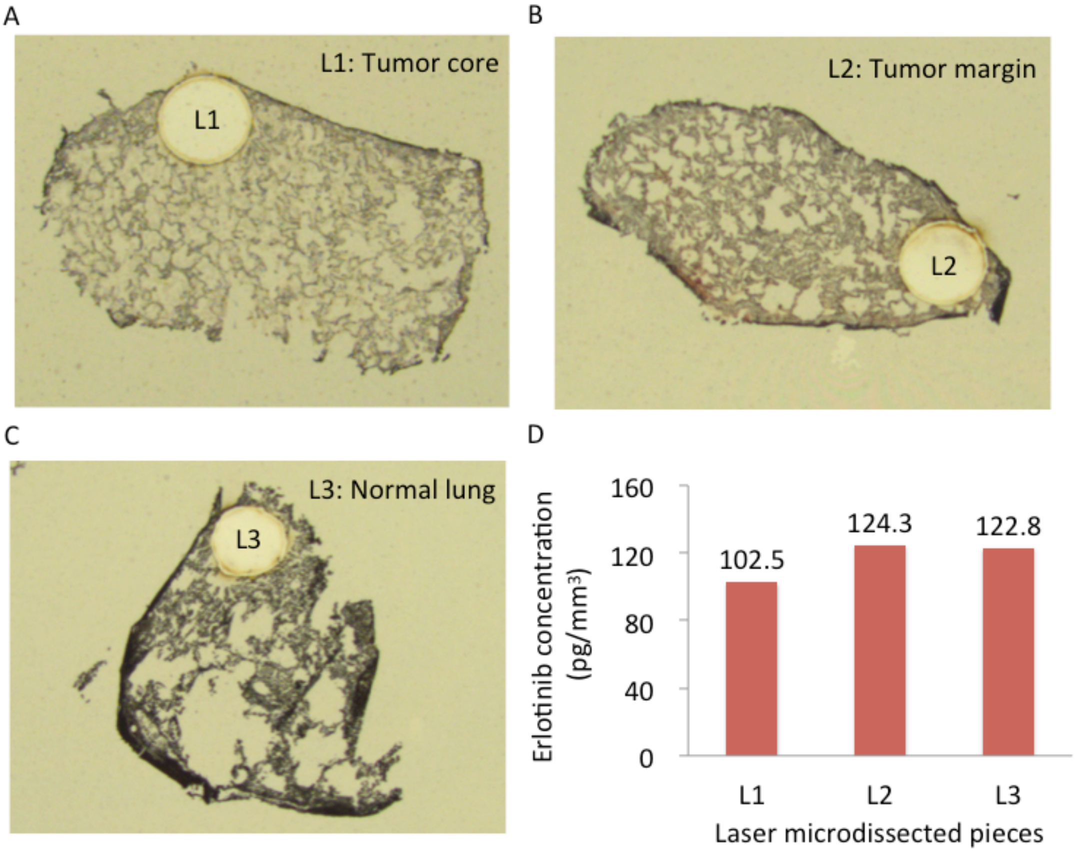

Figure S4. Erlotinib tissue concentrations by liquid chromatography-tandem mass spectrometry analysis in the laser microdissected pieces of the clinical samples

(A, B, C) Serial sections of mass spectrometry images were laser microdissected in the tumor core, tumor margin, and normal lung tissue sections, respectively. (D) Erlotinib concentrations in the laser microdissected pieces by liquid chromatography-tandem mass spectrometry analysis.
