# Supplementary material for: Evaluation of the heterogeneous tissue distribution of erlotinib in lung cancer using matrix-assisted laser desorption ionization mass spectrometry imaging
Source: Sci Rep. 2017 Oct 3;7:12622. doi: 10.1038/s41598-017-13025-8 (PMC5626687; doi:10.1038/s41598-017-13025-8)
Supplement: Supplementary file 2 — Clinical trial protocol [file 41598_2017_13025_MOESM2_ESM.pdf]

High-sensitivity imaging of erlotinib in resected non-small-cell lung cancer

Trial number:

UMIN000009745

Registration date:

Jan 10, 2013

Condition:

Non-small-cell lung cancer

Classification by specialty:

Pneumology, Hematology and clinical oncology

Protocol:

Version 3. September 16, 2012

CONFIDENTIAL

1. Background

The advent of Epidermal growth factor receptor (EGFR) - tyrosine kinase inhibitor (TKI), a molecular targeted agent, has triggered a major shift in the treatment of inoperable or recurrent non-small cell lung cancer (NSCLC). Two phase III clinical trials examining the effect of gefitinib as an initial treatment for EGFR mutation positive cases were conducted in Japan, and the results were reported in 2010 in both cases. In the WJTOG3405 trial, which was a comparative study with cisplatin/ docetaxel, the PFS as the main endpoint was 9.2 months versus 6.3 months (P <0.0001), the response rate was 62.1% versus 32.2%, a significantly good result in the gefitinib group was gotten[1)]. In addition, in the NEJ002 trial, which was a comparative study with carboplatin/ paclitaxel, the significant prolongations at PFS (10.8 months versus 5.4 months, P <0.0001), and the overall survival (30.5 months vs 23.6 Good results have been obtained even in the month) were observed[2)]. Erlotinib, which is the same type as gefitinib, also showed the significant improvement on PFS in a comparative study with carboplatin/ gemcitabine (Optimal test; 13.1 months versus 4.6 months, P <0.0001)[3)]. Based on these results, EGFR-TKI is positioned as a very important molecular targeted agent to be used even after the initial treatment or after the second treatment of EGFR mutation positive NSCLC.

On the other hand, recently molecular imaging technology has made dramatic progress with the development of new equipment and measuring methods. The latest mass spectrometer (Imaging Mass Microscope, Shimadzu, Japan) can directly perform molecular imaging using a laser[4)]. In other words, since there is no need for radiolabeling and there is no need to homogenate the biological sample, it has the advantage that the distribution of the drug at the tissue and cell level is visualized. Actually, distribution analysis of anti-bacterial agent in an animal model transplanted with M. tuberculosis[4)], semi-quantitative mass spectrometry imaging of metabolites of mouse liver transplanted with human colon cancer cells[5)] have been reported. However, there are few reports that mass imaging using anticancer drugs was carried out.

Although it is thought that EGFR-TKI binds to the mutated part of EGFR overexpressed in tumor tissues after oral administration, the pharmacokinetics, such as which part of the tumor tissue the EGFR-TKI exactly distributed, is unknown. Imaging Mass Microscope has a high spatial analysis capability, and it is possible to evaluate a combination of tissue morphology information and mass spectrometry. By analyzing the tumor tissue after oral administration of EGFR-TKI, visualization of the EGFR-TKI distribution at the cellular level is expected to be possible. Here, we planned to study the utility of mass spectrometry

CONFIDENTIAL

imaging using NSCLC surgical specimen of EGFR-TKI patient after oral administration of erlotinib. The purpose of this clinical research is to carry out the world's first mass imaging of EGFR-TKI using an Imaging Mass Microscope that could lead to detailed elucidation of the mechanism of action and tolerance of EGFR-TKI.

2. Ethics and compliance

This study shall be conducted in compliance with the spirit of the Helsinki Declaration (Edinburgh revised edition, 2000) and in accordance with this study protocol.

3. Objective

We perform mass spectrometry imaging of erlotinib using resected non-small cell lung carcinoma after oral administration of erlotinib. We semi-quantitatively identify the presence of erlotinib and its metabolites in tumor tissue/ non-tumor tissue and investigate the mechanism of drug efficacy development.

4. Study procedure

4.1. Oral dosing of erlotinib

Patient diagnosed with EGFR mutation-positive NSCLC and scheduled tumor resection for histology or cytology is registered (N = 1).

Patient receives erlotinib (1 tablet of 150 mg) on the morning of the surgery day (take 6 hours before the start of surgery).

4.2. Measurement of blood erlotinib concentration

Blood collection is performed at the time of tumor tissue removal, and blood levels of erlotinib and its metabolites are measured. Collect 2 ml in a hemogram test tube and separate it into plasma and blood cell components as soon as possible. Separate plasma and blood cells after separation and quickly store at -20 ° C. Saved samples are anonymized and sent to research cooperation facilities (National Cancer Center Research Institute). Measure the erlotinib concentration in plasma by liquid chromatography tandem mass spectrometry[6)].

Sample destination:
Akinobu Hamada, PhD
National Cancer Center Research Institute (NCCRI)
5-1-1 Tsukiji, Chuo-ku, Tokyo, 104-0045 Japan
Phone: +81-3-3547-2511 FAX: +81-3-3545-3567

CONFIDENTIAL

4.3. Record of administration and blood collection

Describe the following items in the records of drug administration status and pharmacokinetics and store them. For the pharmacokinetic record, send it to the analytical institute (National Cancer Center Research Institute) together with the sample.

- Erlotinib administration day and time
- Tumor tissue excision time
- Blood collection time
- Adverse events appearnce

4.4. Handling of excised tumor tissue

Tumor tissues removed by surgery should be frozen by liquid nitrogen as quickly as possible and stored at -80 ° C. The stored tissues are sent to the analytical institute (National Cancer Center Research Institute). The specimens of 1 cube centimeter or more of the marginal part and the center part of the tumor as well as the part considered as normal lung, are sent, respectively.

4.5. Analysis: mass spectrometry imaging of erlotinib

Mass spectrometry imaging of erlotinib in tissues is carried out using an iMScope (Shimadzu, Kyoto, japan).

5. Indication

5.1. Inclusion criteria

- Histologically or cytologically proven diagnosis of Non-small-lung cancer
- EGFR mutation positive
- Stage I–IIIA, operable
- Eastern Cooperative Oncology Group (ECOG) PS 0-1
- Adequate organ and marrow function defined as follows

  WBC ≥ 4,000/μL, ANC ≥ 2,000/μL

  Platelets ≥100,000/mm^3 (100 x 10^9/L)

CONFIDENTIAL

Hemoglobin ≥ 9 g/dL (90 g/L)

Serum creatinine ≤ ULN or estimated creatinine clearance ≥90 ml/min as calculated using the method standard for the institution

Total serum bilirubin ≤ ULN

AST and/or ALT ≤ ULN

PaO2 ≥ 80 mmHg or SpO2 ≥ 95%

- Evidence of a personally signed and dated informed consent document indicating that the patient has been informed of all pertinent aspects of the study

5.2. Exclusion criteria

- Smoking history
- Lung disease such as emphysema, fibrosis, organized by image examination that is not related to cancer
- Obstructive or restrictive pulmonary disorders
- Case with serious complications
- Sever infection
- Severe comorbidities
- Patients who received any chemotherapy or radiotherapy
- Known or possible hypersensitivity to the drug that may increase the risk associated with study participation
- Patients who are pregnant or lactating. Patients of childbearing potential
- Case in which the doctor in charge judged inappropriate

6. Patient Information and consent

6.1. Description using document

Prior to registration of the case in this study, the attending physician will explain sufficiently to the patient and its families deemed appropriate as subjects using the explanatory documents on the following matters. In doing so, give enough time to ask questions and to decide whether to participate in the study.

CONFIDENTIAL

- Purpose and method of this study
- Expected effects and adverse event and its treatment
- Do not receive disadvantages even if you do not agree to participate in the study
- Be able to withdraw this from anytime even after agreeing to participate in the study
- Other necessary matters concerning human rights protection of patients

6.2. Acquisition of consent using documents

After explanation using the explanatory document, when consent based on the patient's free willing is obtained about participation in this study, obtain the signature and consent date of the patient in the consent form integrated with the explanation. The doctor who explained will sign the consent form and state the date of consent. The written agreement will be in three copies, some of it will be stored in the electronic medical record and some will be delivered to the patient.

6.3. Withdraw consent

If document agreement is obtained and then consent is withdrawn, record the date and the reason for withdrawal of consent.

7. Registration, treatment start, progress report and end report

7.1. Registration

If there is a case judged to be able to be registered (a case that satisfies the inclusion criteria and does not fall under the exclusion criteria), the attending physician obtains consent from the document and then contacts the study corresponding investigator.

7.2. Treatment start, progress report, and discontinuation/ termination report

The treatment contents performed in this study is defined as protocol treatment. The doctor in charge of study informs the study responsible doctor as appropriate the initiation, progress report, and completion report of the protocol treatment and always conducts it while taking cooperation.

8. Intervention method

8.1. Protocol drug

Erlotinib (trade name; Tarceva tablet ®)

CONFIDENTIAL

Refer to the package insert for drug handling. Study drugs are purchased from Chugai Pharmaceutical under the research expenses by the Research Director and administered to the subjects.

8.2. Administration of protocol drug and schedule of tumor removal

Erlotinib (Tarceva) is administered according to the schedule described in the Study Procedure section, and the tumor tissue is removed. Tarceba is administered by inversion taking as the guideline after 6 hours of oral administration when it is known that the time of tumor removal is the maximum blood concentration at the time of Tarceva tablet 150 mg single administration oral administration. Regarding adjuvant chemotherapy after surgery, examination will be made according to the results of histopathological examination, not specified.

<i.e. Examination schedule>

On the day of the exam (surgery) (an example in the case of conducting surgery in t he morning)

AM 7: 00 Tarceva tablet ® 150 mg 1 tablet

AM 8:30 Exit to the operating room

AM 9:30 Surgery started

PM 1:00 Removal of tumor tissue, collection of blood for blood concentration

8.3. Monitoring toxicity

Although this study is a single administration of the protocol drug, toxicity monitoring is also carried out just after the tumor extirpation surgery. Adverse events admitted within 2 weeks from erlotinib intake day (surgery day) and its NCI-CTCAE grade are all recorded. In order to grasp the signs of interstitial pneumonia at an early stage, SpO2 monitor and appropriate chest X-ray photograph are carried out for 5 days after surgery. Also, blood tests should be conducted to check for liver function abnormalities as a rough guide for 1 week after administration.

9. Observation items

The study physician performs the following observation and examination and fills out the result in the data sheet and the chart.

9.1. Background: before administration for protocol drug

CONFIDENTIAL

① Patient identification code (registration number)

② Date of document consent acquisition

③ Age (at registration)

④ Sex

⑤ Height

⑥ Body weight

⑦ Medical history

⑧ Complications (excluding accompanying symptoms due to the original disease)

⑨ Drug allergy

⑩ Clinical diagnosis, definitive diagnosis method (tissue diagnosis or cell diagnosis)

⑪ Stage classification (at initial diagnosis), TNM classification

9.2. Blood biochemical examination

Before administration of Tarceva (on the day of surgery or the day before surgery), days 2, 4, 7 after surgery.

Hemoglobin, white blood cell count, leukocyte fraction, number of platelets

Total bilirubin, direct bilirubin, AST, ALT, γ-GTP, LDH, ALP, CRP

9.3. Self symptoms and medical examination findings

Before administration of Tarceva (before surgery), daily until 7th day after surgery

Diarrhea, fever, rash, dyspnea

10. Evaluation of safety

For adverse events occurring during treatment of this protocol (from the day of Tarceva administration to 1 week after surgery), all grade, time of onset, outcome etc. should be indicated on the treatment record sheet. Grade evaluation follows the Japanese translation of NCI Common Toxicity Criteria (NCI - CTC) Version 4.0 JCOG version.

11. Obligation to report adverse events

11.1. Expedited report

11.1.1. Object of expedited report

All of the following adverse events that occurred during this clinical trial are subject to

CONFIDENTIAL

expedited report.

① All deaths that occurred within 7 days from the day of protocol treatment and study drug administration

② Mortality that can not deny the causal relationship with protocol treatment after 7 days from the day of administration of the study drug

③ Serious adverse event not anticipated

④ Acute lung injury

11.1.2. Procedure of expedited report

The doctor in charge of study and the investigator will report according to the following procedure.

① Primary report: The doctor in charge of the study reports the first report to the investigator as soon as possible after knowing the occurrence of the adverse event. In addition, the study physician will prepare as a separate sheet "Detailed case report details (A4 free format)" with detailed information written within 7 days, submit it to the investigator and the department manager in charge. At that time, since we prioritize the transmission of information promptly, there may be places that are undetermined and can not be entered in the report.

② Secondary Report: The investigator will prepare a detailed "Adverse Event Report" as a rule within 15 days from knowing the occurrence of the adverse event and report it to the person in charge of the department concerned and the safety evaluation committee.

③ Additional report: In case of information (eg autopsy report in the case of death) since the secondary report or blank entry in the secondary report, each person in charge of department and safety report to the safety evaluation committee.

CONFIDENTIAL

11.1.3. Definition of severity and expectancy

Among adverse events, the following are serious adverse events.

① Those leading to death

② Threatening life

③ Requiring hospitalization for treatment or extension of hospitalization period

④ Persistent or obvious failure or malfunction

⑤ Those causing birth defects

⑥ Even if the above result is not reached, in a serious event such as subjecting the subject to extreme crisis or requiring treatment so that the result as described above is not reached, or an event having a possibility of leading to these What the physician deems serious

11.2. Regular report

Regarding the following adverse events, a "regular report" is conducted in accordance with the secondary report of the express report.

① Adverse events of Grade 2, Grade 3 that are not anticipated (not listed in "side effects" of drug package insert)

② An adverse event of Grade 3, 4 (as described in "side effects" of drug package insert) expected

③ Other serious medical events (those judged by the attending physician as important information to be shared within the research group)

11.3. Obligations of the study corresponding investigator

11.3.1. Contact to participating facilities

The investigator examines the contents of the "adverse event report", judges the urgency, the importance, the extent of the influence, etc., and notifies the doctor in charge of coping with necessary measures as necessary, and thoroughly notifies it.

11.3.2. Report to the safety evaluation committee

The study corresponding investigator reports to the third party safety evaluation committee the viewpoint and response to the reported adverse event, and requests the review of the opinion of the research director and the validity of the correspondence. The Safety Evaluation Committee reviews and examines the content of the report and recommends to the investigator in writing on future response.

12. Stop and abort study

In case the investigator have found new findings that can be considered a serious problem in continuing the examination and decided that it is necessary to stop the whole study or have been advised by the safety evaluation committee to stop the examination, the investigator will consult with the safety assessment committee. As a result, this test is canceled if judging that

CONFIDENTIAL

the subject's safety can not be secured even though measures such as revision of the test implementation plan are taken.

13. Case accumulation period

We will schedule the 2012 IRB approval ~ March 2014.

14. Protection of patient's personal information

In this study, all patient data and sending samples are identified by case registration number to protect patient's personal information. For the management of data, the connectable anonymous method is used. Also, regarding public announcement of test results, careful consideration is given to the protection of personal information of patients.

15. Research organization

15.1. Corresponding investigator

Yukari Tsubata

Assistant Professor, Division of Medical Oncology & Respiratory Medicine, Department of Internal Medicine, Shimane University Faculty of Medicine

89-1 Enya-cho, Izumo, Shimane, Japan

TEL/FAX 81-853-20-2580

E-mail : ytsubata@med.shimane-u.ac.jp

Takeshi Isobe

Professor, Division of Medical Oncology & Respiratory Medicine, Department of Internal Medicine, Shimane University Faculty of Medicine

89-1 Enya-cho, Izumo, Shimane, Japan

TEL/FAX 81-853-20-2580

E-mail: isobeti@med.shimane-u.ac.jp

Funding Source

Self funding, Division of Medical Oncology & Respiratory Medicine, Department of Internal Medicine, Shimane University Faculty of Medicine

15.2. Cooperation institute

Akinobu Hamada

National Cancer Center Research Institute (NCCRI)

CONFIDENTIAL

5-1-1 Tsukiji, Chuo-ku, Tokyo, 104-0045 Japan

TEL: 81-3-3547-2511 FAX: 81-3-3545-3567

15.3. Safety evaluation committee

Kitaguchi Souichi

Head, Department of Respiratory Medicine, Asa-city Hospital

2-1-1, Kabe-Minami, Asa-Kita-ku, Hiroshima, 731-0293 Japan

TEL/FAX: 81-82-815-5211

16. References

１）Mitsudomi T, Morita S et al : Gefitinib versus cisplatin plus docetaxel in patients with non-small-cell lung cancer harbouring mutations of the epidermal growth factor receptor (WJTOG3405) : an open label, randomized phase 3 trial. Lancet 11: 121-128 (2010)

２）Maemondo M, Inoue A et al : Gefitinib or chemotherapy for non-small-cell lung cancer with mutated EGFR. N Engl J Med 362: 2380-2388 (2010)

３）Zhou C, Wu YL et al : First-line treatment with erlotinib improved progression-free survival in advanced lung cancer. Ann Oncol 21: supple 8; abstr LBA13 (2010)

４）Prideaux B, Dartois V et al : High-sensitivity MALDI-MRM-MS imaging of moxifloxacin distribution in tuberculosis-infected rabbit lungs and granulomatous lesions. Anal Chem 83: 2112-2118 (2011)

５）Ikeda K, Kubo A, Akahoshi N et al: Triacylglycerol/phospholipid molecular species profiling of fatty livers and regenerated non-fatty livers in cystathionine beta-synthase-deficient mice, an animal model for homocysteinemia/homocystinuria. Anal Bioanal Chem 400: 1853-1863 (2011)

６）Lepper ER, Swain SM, Tan AR, Figg WD, Sparreboom A: Liquid-chromatographic determination of erlotinib (OSI-774), an epidermal growth factor receptor tyrosine kinase inhibitor. J Chromatogr B Analyt Technol Biomed Life Sci. 25;796 (1):181-8 (2003)

CONFIDENTIAL
